# *CNR1* Genotype Influences HDL-Cholesterol Response to Change in Dietary Fat Intake

**DOI:** 10.1371/journal.pone.0036166

**Published:** 2012-05-02

**Authors:** Heidi J. Silver, Kevin D. Niswender, Charles D. Keil, Lan Jiang, Qiping Feng, Sally Chiu, Ronald M. Krauss, Russell A. Wilke

**Affiliations:** 1 Division of Gastroenterology, Hepatology and Nutrition, Department of Medicine, Vanderbilt University School of Medicine, Nashville, Tennessee, United States of America; 2 Tennessee Valley Healthcare System, Nashville, Tennessee, United States of America; 3 Division of Diabetes, Endocrinology and Metabolism, Department of Medicine, Vanderbilt University School of Medicine, Nashville Tennessee, United States of America; 4 Center for Human Genetics Research, Department of Molecular Physiology and Biophysics, Vanderbilt University School of Medicine, Nashville, Tennessee, United States of America; 5 Division of Clinical Pharmacology, Department of Medicine, Vanderbilt University School of Medicine, Nashville, Tennessee, United States of America; 6 Children's Hospital Oakland Research Institute, Oakland, California, United States of America; Brigham and Women's Hospital and Harvard Medical School, United States of America

## Abstract

**Background:**

Success in further reducing the burden of cardiovascular disease (CVD) is threatened by the increasing prevalence of obesity-related atherogenic dyslipidemia. HDL-cholesterol (HDL-C) level is inversely correlated with CVD risk; each 1 mg/dl decrease in HDL-C is associated with a 6% reduction in risk. We previously showed that a common *CNR1* haplotype, H3 (frequency 20%), is protective against the reduction in HDL-C that typically accompanies weight gain. In the present study, we extend that observation by reporting the effect of *CNR1* haplotype on HDL-C response to modification of dietary fat intake in weight maintenance and weight loss.

**Methods:**

Six haplotype tagging SNPs that cover the *CNR1* gene locus were genotyped in 590 adults of varying body mass index (cohort 1 is 411 males with BMI 18.5–30.0 kg/m^2^; cohort 2 is 71 females with BMI18.5–30.0 kg/m^2^; and cohort 3 is 108 females with BMI 30–39.9 kg/m^2^). Dietary intakes were modified so that fat intake in the “high fat” condition was 15–20% greater than in the “low fat” condition, and lipid profiles were compared between carriers *versus* noncarriers for each of the five commonly observed *CNR1* haplotypes (H1–H5).

**Results:**

In normal to overweight subjects on eucaloric diets, the H3 haplotype was significantly associated with short-term high fat diet induced changes in HDL-C level in females (carriers 5.9 mg/dl>noncarriers, *p* = 0.007). The H3 haplotype was also significantly associated with HDL-C level after 16 weeks on high fat calorie restricted diet in obese females (carriers 6.8 mg/dl>noncarriers, *p* = 0.009).

**Conclusion:**

Variability within the *CNR1* gene locus contributes to gender-related differences in the HDL-cholesterol response to change in dietary fat intake. Functional characterization of this relationship *in vitro* may offer insights that potentially yield therapeutic guidance targeting dietary macronutrient composition, a direction much needed in the current epidemic of obesity.

## Introduction

Obesity is associated with an atherogenic lipoprotein profile characterized by increased very low-density lipoprotein triglycerides (VLDL-TG), small dense low density lipoprotein cholesterol (LDL-C), and decreased circulating high density lipoprotein cholesterol (HDL-C) [1]. The anti-inflammatory properties of HDL-C and its role in reverse cholesterol transport make it a significant modulator of risk for the development of cardiovascular disease (CVD). As event rate is inversely correlated with HDL-C concentration, each 1 mg/dl decrease in HDL-C is associated with a 6% reduction in CVD risk [2]. Decreased HDL-C is especially important in the current epidemic of obesity as higher body mass index (BMI) is associated with lower HDL-C levels in all racial/ethnic groups [3]. Notably, the prevalence of subthreshold (“at risk”) HDL-C levels has improved three-fold more in men than women (↓5.6% *versus* ↓1.8%) over the past 30 years [4]. In fact, the strongest clinically-relevant demographic predictors of plasma HDL-C concentration are BMI and gender [5], [6].

While modest weight loss of 5–10% improves plasma lipoprotein profile [7] and modifying dietary macronutrient intake can increase HDL-C level [8], scientific and clinical debate continues regarding the importance of the relative amount of fat in a calorically restricted diet [9], [10]. Low-fat (LF) diets can increase circulating VLDL-TG levels, reduce HDL-C, and thus, adversely affect atherogenic dyslipidemia [11], [12]. Although meta-analyses show greater improvements in HDL-C on high fat (HF) diets [13], [14], some studies yield conflicting results [15], [16]. Overall, the lipoprotein response to dietary macronutrient composition is a highly variable and complex trait [11], influenced by BMI [17], gender [18], and individual genetic characteristics [19], [20].

Preclinical and clinical studies targeting the endocannabinoid system (ECS) reveal potent effects on body weight, body fat distribution, and lipoprotein metabolism. The ECS consists primarily of two phospholipid-derived signaling molecules, N-arachidonoylethanolamide (anandamide) and 2-arachidonoylglycerol (2-AG), and two G protein-coupled receptors, CB_1_ and CB_2_ [21]. Most of the metabolic effects of ECS signaling appear to be mediated through CB_1_ receptors in central and peripheral tissues. In randomized clinical trials, CB_1_ receptor blockade resulted in weight loss, with increased HDL-C levels that exceeded the magnitude of change expected from weight loss alone [22], [23]. Collectively, these observations suggest that the ECS regulates lipid homeostasis via peripheral mechanisms that are at least partially independent of weight change.

Consistent with this hypothesis, we previously reported that sequence variation in CB_1_ (gene name *CNR1*) is associated with variability in circulating HDL-C levels independent of BMI [24], [25]. Importantly, a common *CNR1* haplotype, H3, was further protective against the marked reduction in HDL-C that occurs in severe obesity (BMI≥40 kg/m^2^) [25]. However, it remains unknown whether the *CNR1* H3 haplotype influences the effect of dietary fat intake on lipoprotein profiles. We tested this question in adults across a range of BMI who were prescribed various percentages of dietary fat intakes. Herein, we provide evidence that the response of HDL-C to increased dietary fat intake during weight maintenance is strongly influenced by the *CNR1* H3 haplotype in women. We also report that this same H3 haplotype may protect women against low circulating HDL-C during voluntary weight loss.

## Methods

Three distinct cohorts comprising 590 adults who participated in clinical trials designed to modify the percentage of dietary fat consumed were studied. Cohort #1 (normal to overweight males) and cohort #2 (normal to overweight females) were convenience samples from prior trials using eucaloric diets designed for weight maintenance. Because we had previously found an association between *CNR1* and HDL-C in a population sample that was predominantly female [26], [27], we enrolled more males (cohort #1) than females (cohort #2) in the present study (at a ratio of 4∶1) to assure that we would not be missing a significant relationship in males. As the strength of the genotype-phenotype associations in cohorts #1 & #2 were consistent with our prior work, cohort #3 consisted of a sample of obese females on a calorically restricted diet, which not only broadened our range of BMI in females to better match current population demographics, but also allowed investigation of the effects of dietary fat intake within the context of weight loss.

For all subjects, the amount of fat prescribed in the “high fat” (HF) conditions were 15% higher than in the “low fat” (LF) conditions. Dietary intakes were assessed by Registered Dietitians (RDs) from 4-day food diaries and 24-hour diet recalls using multi-pass methodology [28]. Energy and macronutrient intake analysis was performed using Nutrition Data System for Research (NDS, Nutrition Coordinating Center, University of Minnesota, Minneapolis). Written informed consent was obtained and study protocols were approved by the Institutional Review Boards of Children's Hospital and Research Center of Oakland and the E.O. Lawrence Berkeley National Laboratory at the University of California for cohorts #1 & #2 and the Vanderbilt University Medical Center for cohort #3.

### Cohort #1: Males on Eucaloric Diet

Males age 21–60 years with BMI 18.9–30 kg/m^2^ who had participated in one of four trials [26], [29], [30], [31], [32] were included if they had no active chronic disease, did not take lipid altering medications, and were nonsmokers. Males were randomly assigned in a crossover design to eucaloric HF diet (prescribed as 35% fat [∼18% saturated, 11% monounsaturated, 6% polyunsaturated], 50% carbohydrate, 15% protein) and LF diet (prescribed as 20% fat [∼5% saturated, 11% monounsaturated, 4% polyunsaturated], 65% carbohydrate [half simple and half complex], 15% protein) for 6 weeks each. Cholesterol content of the prescribed diets was 125 mg/1000 kcal and fiber content was 5 g/1000 kcal. Subjects maintained habitual physical activity and weighed themselves daily. RDs adjusted menu energy content when necessary to maintain weight. Diet compliance was assessed from 4-day weighed food intake records and daily recording of menu items consumed. Height, weight and venous blood samples were collected at baseline and end of study in an overnight fasted state. Plasma total cholesterol and triglyceride levels were determined by enzymatic procedures on a Gilford Impact 400E or Express 550 Plus analyzer (Ciba Corning, Oberlin, OH). HDL-C was measured after dextran sulfate precipitation of plasma and LDL-C was calculated from the Friedewald formula [33].

### Cohort #2: Females on Eucaloric Diet

Premenopausal females age 21–50 years with BMI 18.9–30 kg/m^2^ were included if they had no active chronic disease, did not take lipid altering medications, were nonsmokers, and were not pregnant, breastfeeding or taking oral contraceptives [27]. Although not randomly crossed over, females were prescribed similar eucaloric HF and LF diets as the males in cohort #1 (above) for 8 week periods. The same procedures as described for cohort #1 were followed for compliance and outcome measurements.

### Cohort #3: Females on Calorie Restricted Diet

Premenopausal females age 21–50 years with BMI 30–39.9 kg/m^2^ were included if they had no active chronic disease, did not take medications for lipid metabolism, were nonsmokers, and were not pregnant, breastfeeding or taking oral contraceptives. After habituation on the lower fat diet condition (35% fat), subjects were prescribed a 15% calorically restricted HF diet (50% fat [1/3 saturated, 1/3 monounsaturated, 1/3 polyunsaturated], 30% carbohydrate [half simple and half complex], 20% protein) for 16 weeks. Cholesterol content of the HF diet was 150 mg/1000 kcal and fiber content was 7 g/1000 kcal. Standard assays were performed for triglyceride, total cholesterol, LDL-C and HDL-C by selective enzymatic hydrolysis at the Vanderbilt Department of Pathology Clinical Laboratory. Serum glucose was quantified by colorimetric timed endpoint method and serum insulin by chemiluminescent immunoassay. The homeostasis model assessment of insulin resistance (HOMA-IR) was calculated as [fasting glucose (mM) x fasting insulin (mU/L)]/22.5 [34].

### Genotyping

**Figure 1:**
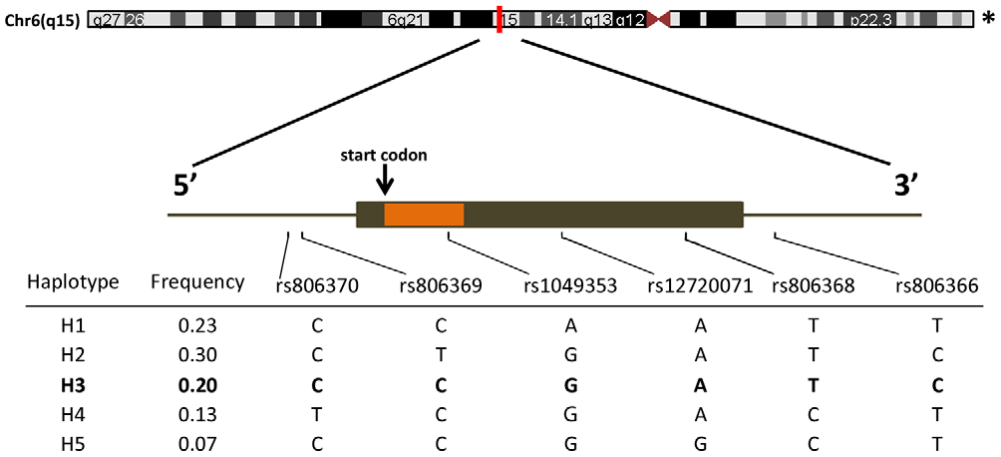
The *CNR1* gene: chromosomal location, tagSNPs, haplotype structure and alleles. *The CNR1 gene locus covers ∼15 kb on Chromosome 6 (chromosome 6 is shown in the reverse orientation, with arm q on the left). Six haplotype tagging SNPs were genotyped, based upon our prior work [25], and reference sequence numbers for the tag SNPs are presented. These SNPs are inherited in combination (haplotypes).

Genomic DNA was extracted from whole blood according to standard procedures. Based on our prior work [24], [25], we tagged the entire *CNR1* gene plus 5 kilobases (kb) upstream and 5 kb downstream using Haploview (http://www.broad.mit.edu/mpg/haploview/) for linkage disequilibrium analysis [35] and selection of tagSNPs. Gene locus, haplotype structure and allele frequencies of the tagSNPs covering the variability within our locus of interest (Chromosome 6, physical position 88, 901, 306–88, 916, 775) are shown in Figure 1. These six tagSNPs include two promoter SNPs (rs806370, rs806369), one coding SNP (rs1049353), two SNPs in the 3′ untranslated region (3′-UTR: rs12720071, rs806368), and one SNP distal to the polyA tail (rs806366). The tagSNPs were genotyped for cohorts #1 & #2 using multiplex polymerase chain-reaction (PCR), single base primer extension (SBE), and generation of mass spectra (Sequenom, San Diego, CA). For cohort #3, genotyping was conducted using commercial Taqman Allelic discrimination assays (Applied Biosystems, Inc., Foster City, CA) [36]. Each assay contained two Taqman probes uniquely labeled to bind separate (major *versus* minor) alleles.

### Statistical Analysis

Statistical analyses were conducted using SPSS version 17 (SPSS Inc., Chicago, IL), JMP version 7.0 (SAS Institute Inc., Cary, NC) and PLINK genetic analyses toolset version 1.07 (http://pngu.mgh.harvard.edu/~purcell/plink) [37]. Data are expressed as means and standard deviations unless otherwise noted. Baseline lipid variables among cohorts were assessed using the Kruskal-Wallis test and relationships between demographics and lipids were examined using Spearman Rho correlations. Minor allele frequency and Hardy-Weinberg (H-W) equilibrium statistics were calculated for each SNP. As we have previously demonstrated that haplotypes provide greater power than single SNPs for identifying associations related to *CNR1* [25], we performed haplotype analyses based on a dominant model (carrier *versus* noncarrier). Five *CNR1* haplotypes (Figure 1) were identified at frequency over 5%. From 5′ to 3′, they are CCAATT (23%), CTGATC (30%), CCGATC (20%), TCGACT (13%), CCGGCT (7%). These five are the most common *CNR1* haplotypes found in the Centre d'Etude du Polymorphisme Humain (CEPH) cohort within the International HapMap Consortium and in our prior cohorts [24]. Although the tagging SNPs were, by definition, in tight linkage dysequilibrium, we corrected for multiple hypothesis testing of the five haplotypes [38] and considered results statistically significant only with *p* value<0.01.

For cohorts #1 and #2, the five *CNR1* haplotypes were estimated using PHASE software. As expected, the same five haplotypes were observed in cohort #3. However, the greater racial diversity of this cohort introduced more structural variability at the *CNR1* gene locus, and thus, we identified two novel *CNR1* haplotypes (CCGGTT, frequency 0.7%; and CCGATT, frequency 2%) in the African American (AA) females (data not presented). Importantly, these two new haplotypes were not significantly associated with HDL-C levels. For cohort #3, haplotypes were estimated by applying standard E-M algorithms located in the **–hap** option of PLINK.

All genotype-phenotype association analyses were performed using the five *CNR1* haplotypes. In cohorts #1 and #2, linear regression models were created to examine relationships between haplotypes and lipid variables. Data were adjusted for age and baseline BMI. To be consistent with this approach, for cohort #3 a linear regression model was applied to test each haplotype (*versus* all other haplotypes) using the **–hap-linear** option in PLINK. For cohort #3, which was obese by definition, baseline BMI and HOMA-IR were significantly associated with baseline serum HDL-C, and thus, included as covariates in the model using the **–covar** option. This approach allowed us to replicate our initial findings from cohorts #1 &#2 in a second independent cohort (cohort #3) to reduce the likelihood of a spurious association between *CNR1* and HDL-C levels.

## Results

### Subject Characteristics

The combined sample included 411 males and 179 females with a mean age of 39.9±4.0 years (Table 1). As defined by eligibility criteria, subjects in cohorts #1 & #2 were normal to overweight (mean BMI 24.8±1.7 kg/m^2^), while cohort #3 subjects were obese (mean BMI 34.7±2.7 kg/m^2^). Ninety percent of subjects in cohorts #1 & #2 self-identified as being of European ancestry (EA). In cohort #3, 75 (69%) females self-identified as EA and 33 (31%) as AA. Baseline TG, total cholesterol, LDL-C and HDL-C were similar across cohorts. There were no significant associations between minor allele frequency and diet adherence (data not presented).

**Table 1:** Baseline characteristics of study subjects (n = 590).

Characteristics	Cohort #1	Cohort #2	Cohort #3
Sample Size	411	71	108
Male/Female	Male	Female	Female
Age, mean (SD), y	45.0 (11.5)	38.2 (6.5)	37.5 (0.7)
BMI*, mean (SD)	25.4 (3.0)	23.2 (3.1)	36.7 (1.1)
Total cholesterol, mean (SD), mg/dL	197.9 (35.1)	186.3 (31.0)	168.1 (29.3)
Triglycerides, mean (SD), mg/dL	129.6 (77.4)	80.7 (44.2)	85.1 (48.2)
HDL-C, mean (SD), mg/dL	46.6 (12.2)	60.3 (11.3)	48.9 (12.8)
LDL-C, mean (SD), mg/dL	125.7 (30.4)	109.8 (29.1)	102.1 (25.0)

Abbreviations: SD, standard deviation; BMI, body mass index; HDL-C, high density lipoprotein cholesterol; LDL-C, low-density lipoprotein cholesterol.

* Calculated as weight in kilograms divided by height in meters squared.

### Cohort #1

As expected with eucaloric diets, no significant changes occurred in body weight in cohort #1 subjects. In these normal to overweight males, HF diet significantly decreased TG (−30.2±63.6 mg/dl, *p*<0.001), increased total cholesterol (+12.0±21.9 mg/dl, *p*<0.001), increased LDL-C (+11.5±20.6 mg/dl, *p*<0.001) and increased HDL-C (+6.4±5.7 mg/dl, *p*<0.001). Consistent with previous report in a predominantly female cohort [24], the reduction in TG was significantly greater in male noncarriers of the *CNR1* H4 haplotype compared to male carriers (at least one copy), *p* = 0.005. Although prior work also indicated that the *CNR1* H3 haplotype was associated with HDL-C in both genders, in the present study H3 was not significantly associated with HDL-C response to high fat diet in normal to overweight males (Table 2); male carriers of the H3 haplotype had only a 1.3 mg/dl higher HDL-C level (from 40.5±9.2 to 47.8±11.4 mg/dl) than male noncarriers (from 40.1±9.4 to 46.0±11.5 mg/dl).

**Table 2:** Effect of haplotype on triglycerides, LDL-cholesterol and HDL-cholesterol in 411 normal to overweight males (Cohort #1) on eucaloric diets.

	Triglyceride, mg/dl (SD)			LDL Cholesterol, mg/dl (SD)			HDL Cholesterol, mg/dl (SD)		
Haplotype	Non-Carrier	Carrier	*P*-Value	Non-Carrier	Carrier	*P*-Value	Non-Carrier	Carrier	*P*-Value
H1									
Sample Size	241	170		241	170		241	170	
LF	137.4 (100.2)	145.5 (91.4)	0.81	118.0 (32.7)	109.6 (30.8)	0.007	40.9 (9.0)	39.2 (9.7)	0.29
HF	112.3 (99.7)	108.2 (72.9)	0.19	129.3 (35.1)	121.4 (34.8)	0.02	47.2 (10.9)	45.7 (12.3)	0.65
Difference	−25.1 (61.7)	−37.4 (65.7)	0.09	11.3 (19.5)	11.8 (22.2)	0.72	6.3 (5.5)	6.5 (6.0)	0.45
H2									
Sample Size	191	220		191	220		191	220	
LF	146.3 (107.3)	135.9 (86.3)	0.67	112.7 (32.4)	116.1 (32.0)	0.19	39.7 (9.2)	40.6 (9.4)	0.51
HF	121.0 (117.2)	101.6 (54.0)	0.21	122.7 (34.1)	128.9 (35.8)	0.04	46.0 (11.8)	47.1 (11.2)	0.50
Difference	−25.3 (67.9)	−34.3 (59.4)	0.20	10.0 (21.1)	12.8 (20.2)	0.10	6.3 (5.8)	6.5 (5.6)	0.77
H3									
Sample Size	284	127		284	127		284	127	
LF	143.6 (95.6)	134.5 (99.0)	0.14	113.8 (31.1)	116.2 (34.5)	0.60	40.1 (9.4)	40.5 (9.2)	0.77
HF	111.6 (94.6)	108.3 (77.5)	0.42	126.4 (34.3)	125.2 (37.1)	0.56	46 (11.5)	47.8 (11.4)	0.16
Difference	−31.9 (65.0)	−26.2 (60.4)	0.58	12.6 (20.5)	9.0 (20.7)	0.05	6.0 (5.7)	7.3 (5.6)	0.03
H4									
Sample Size	303	108		303	108		303	108	
LF	139.9 (91.4)	143.2 (110.4)	0.97	114.4 (32.8)	115.0 (30.3)	0.65	39.8 (9.4)	41.2 (9.3)	0.20
HF	104.6 (62.2)	127.4 (139.9)	0.11	126.9 (35.8)	123.7 (33.2)	0.13	46.4 (11.2)	47.0 (12.2)	0.76
Difference	−35.3 (60.4)	−15.8 (70.2)	0.005	12.5 (21.0)	8.7 (19.5)	0.06	6.6 (5.6)	5.8 (6.0)	0.17
H5									
Sample Size	328	63		328	63		328	63	
LF	142.0 (98.2)	133.7 (88.2)	0.60	113.7 (31.7)	119.2 (34.2)	0.22	40.2 (9.5)	40.2 (8.6)	0.56
HF	112.7 (94.1)	98.8 (57.7)	0.49	124.9 (35.1)	132.3 (35.2)	0.14	46.7 (11.6)	46.0 (10.8)	0.27
Difference	−29.3 (65.7)	−34.9 (50.4)	0.44	11.2 (20.4)	13.1 (21.9)	0.56	6.5 (5.7)	5.7 (5.4)	0.23

Abbreviations: LF, low fat diet; HF, high fat diet.

P-values are presented after adjusting for age and baseline BMI; statistical significance level established at α = 0.01.

### Cohort #2

**Figure 2:**
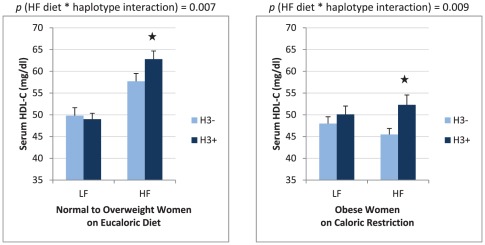
Effect of H3 haplotype on serum HDL-cholesterol levels in women. Values shown are mean+standard error. In normal to overweight females, changing from low fat (LF) to high fat (HF) diet increased HDL-C from 49.0±8.0 mg/dl to 62.8±11.2 mg/dl in H3 haplotype carriers and from 49.8±10.9 mg/dl to 57.7±10.9 mg/dl in H3 non-carriers. In obese females, calorically restricted (−15% kcal) HF diet increased HDL-C from 50.11±11.60 mg/dl to 52.29±10.38 mg/dl in H3 haplotype carriers and decreased HDL-C from 47.99±12.90 mg/dl to 45.49±8.95 mg/dl in H3 non-carriers.

Like the males in cohort #1, females in cohort #2 experienced no significant changes in body weight. In this cohort, HF diet significantly decreased TG (−17.5±34.1 mg/dl, *p*<0.001), increased total cholesterol (+12.2±19.8 mg/dl, *p*<0.001), increased LDL-C (+4.8±17.0 mg/dl, *p* = 0.02) and increased HDL-C (+10.9±8.7 mg/dl, *p*<0.001). In contrast to the males in cohort #1, the *CNR1* H3 haplotype was strongly associated (*p* = 0.007) with high fat diet induced changes in serum HDL-C levels in these normal to overweight females (Table 3). The increase in HDL-C level after HF diet in normal to overweight female carriers of the H3 haplotype (from 49±8 to 62.8±11.2 mg/dl) was 5.9 mg/dl greater than the increase in noncarriers (from 49.8±10.9 to 57.7±10.9 mg/dl) (Figure 2a).

**Table 3:** Effect of haplotype on triglycerides, LDL-cholesterol and HDL-cholesterol in 71 normal to overweight females (cohort #2) on eucaloric diet.

	Triglyceride, mg/dl (SD)			LDL Cholesterol, mg/dl (SD)			HDL Cholesterol, mg/dl (SD)		
Haplotype	Non-Carrier	Carrier	*P*-Value	Non-Carrier	Carrier	*P*-Value	Non-Carrier	Carrier	*P*-Value
H1									
Sample Size	42	29		42	29		42	29	
LF	93.5 (47.5)	105.0 (47.4)	0.18	104.5 (25.5)	105.8 (39.7)	0.91	48.3 (9.8)	50.9 (8.8)	0.26
HF	74.1 (37.1)	90.2 (52.0)	0.08	110.5 (28.3)	108.7 (30.6)	0.82	60.5 (11.5)	59.9 (11.2)	0.74
Difference	−19.4 (32.7)	−14.8 (36.3)	0.53	6.1 (15.7)	2.9 (18.8)	0.56	12.2 (8.1)	9.0 (9.2)	0.14
H2									
Sample Size	42	29		42	29		42	29	
LF	100.9 (54.1)	94.3 (36.4)	0.75	104 (34.5)	106.5 (28.0)	0.71	49.5 (8.6)	49.2 (10.8)	0.89
HF	86.5 (51.9)	72.3 (28.4)	0.31	108.7 (28.5)	111.4 (30.4)	0.68	59.8 (10.1)	61 (12.9)	0.68
Difference	−14.4 (35.5)	−22.0 (32.0)	0.37	4.7 (15.0)	4.9 (19.8)	0.97	10.3 (7.7)	11.7 (10.0)	0.52
H3									
Sample Size	35	36		35	36		35	36	
LF	107.3 (54.9)	89.4 (37.7)	0.61	108.1 (37.7)	102 (25.0)	0.93	49.8 (10.9)	49.0 (8.0)	0.13
HF	88.9 (53.2)	72.7 (32.0)	0.95	108.9 (32.4)	110.6 (25.9)	0.42	57.7 (10.9)	62.8 (11.2)	0.33
Difference	−18.4 (32.1)	−16.7 (36.3)	0.60	0.8 (19.2)	8.6 (13.8)	0.11	7.9 (6.2)	13.8 (9.7)	0.007
H4									
Sample Size	51	20		51	20		51	20	
LF	91.0 (35.2)	116.5 (67.5)	0.81	101.5 (26.7)	113.9 (41.7)	0.55	50.1 (9.3)	47.6 (9.8)	0.78
HF	71.2 (27.5)	104.9 (65.9)	0.58	106.8 (26.1)	117.4 (35.2)	0.44	62 (11.6)	55.8 (9.4)	0.37
Difference	−19.8 (33.2)	−11.7 (36.3)	0.57	5.3 (15.2)	3.5 (21.3)	0.77	12 (9.7)	8.2 (4.3)	0.20
H5									
Sample Size	68	3		68	3		68	3	
LF	97.6 (47.3)	112.7 (60.9)	0.33	105.3 (31.2)	98 (52.4)	0.97	49.4 (9.5)	48.3 (10.1)	0.54
HF	81.1 (45.0)	71.7 (14.4)	0.61	110.2 (29.0)	101.7 (37.1)	0.78	60.4 (11.4)	57.7 (9.0)	0.36
Difference	−16.5 (33.5)	−41.0 (47.1)	0.25	4.8 (17.2)	3.7 (15.5)	0.58	11.0 (8.9)	9.3 (1.2)	0.68

Abbreviations: LF, low fat diet; HF, high fat diet.

P-values are presented after adjusting for age and baseline BMI; statistical significance level established at α = 0.01.

### Cohort #3

The obese females experienced a significant weight loss of 6.2±3.5 kg over the 16-week intervention (*p*<0.001) due to the 15% caloric restriction prescribed in the HF diet condition. Overall, cohort #3 experienced significantly decreased BMI (−2.5±1.5 units, *p*<0.001), total cholesterol (−9.6±19.2 mg/dl, *p*<0.001), LDL-C (−8.0±17.6 mg/dl, *p*<0.001), plasma insulin (−2.03±4.79 mg/dl, *p* = 0.001), and had non-significant changes in TG (−5.58±33.6 mg/dl, *p* = 0.178), HDL-C (0.30±7.90 mg/dl, p = 0.758), and plasma glucose (−1.16±10.3 mg/dl, *p* = 0.360). As with normal to overweight females, the *CNR1* H3 haplotype was strongly associated with serum HDL-C levels in obese females, *p* = 0.009 (Table 4). After 16 weeks of high fat diet, obese female carriers of the H3 haplotype had significantly higher HDL-C levels (52.3±2.3 mg/dl) than noncarriers (45.5±1.4 mg/dl) (Figure 2b). In re-testing the EA obese females alone, the H3 haplotype remained significantly associated (*p* = 0.005) with HDL-C level, and thus, the relationship was independent of race. The relationship between H3 haplotype and HDL-C was also independent of circulating TG level (p = 0.06 for the association between H3 haplotype and TG/HDL-C ratio).

**Table 4:** Effect of haplotype on triglycerides, LDL-cholesterol and HDL-cholesterol in 108 obese females (cohort #3) on calorie restricted diet.^a^

		Triglyceride, mg/dl (SD)			LDL Cholesterol, mg/dl (SD)			HDL Cholesterol, mg/dl (SD)		
Haplotype	Sample Size^b^	Non-Carrier	Carrier	*P*-Value^c^	Non-Carrier	Carrier	*P*-Value^b^	Non-Carrier	Carrier	*P*-Value^c^
H1										
LF	66/40	78.6 (42.6)	96.8 (55.8)	0.06	98.7 (23.8)	106.6 (25.9)	0.11	50.3 (11.8)	46.2 (10.8)	0.10
HF	39/25	75.6 (39.8)	78.2 (39.7)	0.80	98.8 (21.6)	94.7 (23.7)	0.47	49.1 (10.0)	45.6 (9.6)	0.17
H2										
LF	54/52	82.5 (51.7)	88.6 (45.4)	0.52	98.9 (25.1)	104.6 (24.4)	0.24	49.8 (11.9)	47.6 (13.1)	0.38
HF	31/33	68.1 (37.8)	84.6 (39.9)	0.09	93.3 (23.2)	100.6 (21.4)	0.18	48.9 (9.8)	46.6 (10.0)	0.37
H3										
LF	69/37	89.2 (53.3)	78.5 (37.8)	0.28	102.6 (24.5)	99.9 (25.6)	0.60	48.0 (12.9)	50.1 (11.6)	0.41
HF	43/21	79.6 (41.2)	70.5 (35.9)	0.39	100.8 (20.6)	89.3 (24.6)	0.05	45.5 (8.9)	52.3 (10.4)	0.009
H4										
LF	86/20	86.4 (50.2)	81.4 (41.7)	0.68	103.7 (25.0)	93.1 (22.6)	0.08	48.9 (12.6)	48.1 (12.1)	0.80
HF	52/12	76.9 (40.3)	75.3 (37.5)	0.90	97.2 (22.8)	97.1 (21.4)	0.99	48.8 (10.4)	43.2 (6.0)	0.08
H5										
LF	96/10	87.3 (50.0)	68.0 (27.3)	0.23	102.1 (25.1)	97.9 (22.4)	0.61	48.9 (11.8)	47.1 (18.0)	0.67
HF	58/6	75.6 (38.2)	86.5 (53.7)	0.52	96.7 (22.9)	102.2 (17.6)	0.58	47.9 (9.8)	46.2 (11.9)	0.69

Abbreviations: LF, low fat diet; HF, high fat diet; TG, triglycerides; HDL-C, high density lipoprotein cholesterol.

a Number of Non Carriers/number of Carriers.

b Statistical significance set at α = 0.01.

c Subjects were prescribed high fat diet within the context of caloric restriction for weight loss, however, the effects observed between haplotype and HDL-C was independent of weight loss.

## Discussion

The present findings demonstrate that variability in the *CNR1* gene influences HDL-C response to changes in dietary fat intake. In normal to overweight adults on eucaloric diets, subjects expressing the *CNR1* H3 haplotype had a more robust increase in HDL-C level during high fat intake and the magnitude of the effect of the H3 haplotype on HDL-C was four-fold greater in females compared to males. We observed a similar response to high fat intake in females with class I and II obesity who were prescribed caloric restriction. After 16 weeks of high fat diet, obese female carriers of the H3 haplotype had HDL-C levels that were ∼7 mg/dl greater than noncarriers (52.3±2.3 mg/dl in carriers *versus* 45.5±1.4 mg/dl in noncarriers, *p* = 0.009). The current findings are consistent with prior evidence that the *CNR1* H3 haplotype was strongly associated with HDL-C levels in individuals with class III obesity [25].

It is important to recognize that the frequency of the H3 haplotype does not differ by gender and is common (20% frequency) in the general population. Given that women disproportionately seek dietary approaches for weight loss [39], along with the increasing recognition that women are at significant risk for cardiovascular disease - particularly in the current obesogenic environment, it is imperative to understand factors that determine favorable lipoprotein responses to dietary weight loss interventions. Prior investigation indicates that women may be more sensitive to diet induced changes in HDL-C during weight loss than men [40]. For example, a recent comparison of popular weight loss diets showed women had markedly increased HDL-C on 50% dietary fat intake (Atkins diet: ∼5 mg/dl increase in HDL-C), moderately increased HDL-C on 30% fat intake (Zone and Learn diets: ∼2.5 mg/dl increase in HDL-C) and no change in HDL-C on 10% fat intake (Ornish diet) [41]. The gender distinction observed in HDL-C response to popular weight loss diets may in part be attributable to underlying variability in the *CNR1* gene.

The specific physiological mechanism linking the *CNR1* gene product to lipid homeostasis is not completely understood. The CB_1_ receptor modulates food intake through an effect on central neuronal circuits [42]. Central CB_1_ receptor signaling is also known to directly influence peripheral metabolic processes involved in VLDL-TG secretion and clearance [43], [44]. Although changes in the abundance of VLDL could alter circulating HDL-C levels, by shifting the flux of free fatty acids between larger and smaller lipoproteins [45], our data do not support such a conclusion. In the present study, it appears that *CNR1* modulated HDL-C response to high fat diet independent of BMI and fasting TG level. Peripherally, the CB_1_ receptor is expressed in the liver [46], pancreas, gastrointestinal tract [47] and adipose tissue [48]. HDL particle composition, and therefore HDL clearance rate, is modified within these tissues through the action of lipolytic enzymes [49]. Studies conducted using conditional (hepatic) CB_1_ knockout mice have shown that the CB_1_ receptor directly regulates hepatic lipid metabolism [50]. Lipoprotein lipase (LPL) and hepatic lipase (HL) convert larger HDL particles into smaller HDL remnants, resulting in HDL particles that are cleared more rapidly from the circulation. Endothelial lipase (EL) is also a strong predictor of circulating HDL-C concentration [51], [52], and EL expression is modulated by adipocytokines [53]. Because CB_1_ antagonists modulate the synthesis and secretion of adiponectin [54] and pro-inflammatory cytokines like TNFα [55], [56], genetic variability in *CNR1* may be affecting HDL-C level through a mechanism involving its intravascular modeling. While our study was limited to assessment of HDL-C concentration, future investigation is needed to address more mechanistic hypotheses regarding the distribution of particle subfraction and changes in inflammatory biomarkers within the HDL proteome. In addition, large datasets are needed to condition the genetic effect of *CNR1* on LPL, HL and EL, as well as known functional variants in the ATP-binding cassette transporter A1 (ABCA1), cholesteryl ester transfer protein (CETP), and scavenger receptor class B type 1 (SRB1). While all are known to influence HDL-C level [5], [51], [57], combinatorial models assessing interactions [58] will require targeted genotyping within cohorts of enormous size.

As these studies proceed, it is important to recognize that the causative allele linking *CNR1* to HDL-C level remains undetermined. It is likely that the H3 haplotype examined in the present study is only in partial linkage (ie, D′<1.0) with the actual functional allele, a group of common and rare variants with the capacity to alter *CNR1* gene function [59], [60]. To fully characterize these variants, future investigation must target deep re-sequencing of the *CNR1* gene in individuals expressing H3, from cohorts of racially diverse populations enriched for the traits of interest. In conclusion, we have demonstrated that the common H3 haplotype at the *CNR1* gene locus potently influences HDL-C response to change in dietary fat intake in a gender-specific manner. Functional characterization of this relationship may further our understanding of the biology underlying lipid homeostasis, and potentially yield therapeutic guidance to improve public health by optimizing dietary macronutrient modifications, a direction much needed in the current epidemic of obesity.
